# Effect of external beam radiation therapy versus transcatheter arterial chemoembolization for non-diffuse hepatocellular carcinoma (≥ 5 cm): a multicenter experience over a ten-year period

**DOI:** 10.3389/fimmu.2023.1265959

**Published:** 2023-09-25

**Authors:** Ke Su, Fei Wang, Xueting Li, Hao Chi, Jianwen Zhang, Kun He, Zhaoyang Wang, Lianbin Wen, Yanqiong Song, Jiali Chen, Zhenying Wu, Yi Jiang, Han Li, Tao Gu, Chenjie Wang, Yaqi Li, Mengxiang Liu, Qulian Guo, Ke Xu, Lu Guo, Yunwei Han

**Affiliations:** ^1^ Department of Oncology, The Affiliated Hospital of Southwest Medical University, Luzhou, China; ^2^ Department of Radiation Oncology, National Cancer Center/National Clinical Research Center for Cancer/Cancer Hospital, Chinese Academy of Medical Sciences and Peking Union Medical College, Beijing, China; ^3^ Department of General Surgery, Luxian People’s Hospital, Luzhou, China; ^4^ Department of Oncology, 363 Hospital, Chengdu, China; ^5^ Clinical Research Institute, The Affiliated Hospital of Southwest Medical University, Luzhou, China; ^6^ Department of Medical Imaging, Southwest Medical University, Luzhou, China; ^7^ Department of Geriatric Cardiology, Sichuan Academy of Medical Sciences & Sichuan Provincial People’s Hospital, Chengdu, China; ^8^ Department of Radiotherapy, Sichuan Cancer Hospital & Institute, Sichuan Cancer Center, School of Medicine, University of Electronic Science and Technology of China, Chengdu, China; ^9^ School of Medicine, University of Electronic Science and Technology of China, Chengdu, China; ^10^ School of Humanities and Management, Southwest Medical University, Luzhou, China; ^11^ Department of Paediatrics, The Affiliated Hospital of Southwest Medical University, Luzhou, China; ^12^ Department of Oncology, Chongqing General Hospital, Chongqing, China; ^13^ Department of Ophthalmology, The Affiliated Hospital of Southwest Medical University, Luzhou, China

**Keywords:** external beam radiation therapy, transcatheter arterial chemoembolization, hepatocellular carcinoma, PD-1 inhibitors, targeted drugs

## Abstract

**Background:**

The optimal local treatment for HCC with tumor diameter ≥ 5 cm is not well established. This research evaluated the effectiveness of external beam radiation therapy (EBRT) versus transcatheter arterial chemoembolization (TACE) for HCC with tumor diameter ≥ 5 cm.

**Methods:**

A total of 1210 HCC patients were enrolled in this study, including 302 and 908 patients that received EBRT and TACE, respectively. Propensity score matching (PSM) was used to identify patient pairs with similar baseline characteristics. Overall survival (OS) was the primary study endpoint.

**Results:**

We identified 428 patients using 1:1 PSM for survival comparison. Compared with the TACE group, the EBRT group had a significantly longer median OS (mOS) before (14.9 vs. 12.3 months, *p* = 0.0085) and after (16.8 vs. 11.4 months, *p* = 0.0026) matching. In the subgroup analysis, compared with the TACE group, the EBRT group had a significantly longer mOS for HCC with tumor diameters of 5-7 cm (34.1 vs. 14.3 months, p = 0.04) and 7-10 cm (34.4 vs. 10 months, *p* = 0.00065), whereas for HCC with tumor diameters ≥ 10 cm, no significant difference in mOS was observed (11.2 vs. 11.2 months, *p* = 0.83). In addition, the multivariable Cox analysis showed that Child-A, alkaline phosphatase < 125 U/L, and EBRT were independent prognostic indicators for longer survival.

**Conclusion:**

EBRT is more effective than TACE as the primary local treatment for HCC with tumor diameter ≥ 5 cm, especially for HCC with tumor diameter of 5-10 cm.

## 1 Introduction

Hepatocellular carcinoma (HCC) is a relatively common malignancy with a high mortality rate (1). Currently, despite advances in early diagnostic techniques for HCC, most patients are still diagnosed in the advanced stage, which results in poor diagnosis. The median overall survival (mOS) for patients without active treatment is only 4 months (2). Fortunately, various therapies, such as immunotherapy, targeted therapy, radiofrequency ablation (RFA), radiotherapy, and transcatheter arterial chemoembolization (TACE), have been recently developed and proven to prolong the survival of HCC (3–6).

Currently, programmed death 1 (PD-1) inhibitors are showing great promise in the treatment of cancer (7–9). Atezolizumab + bevacizumab has become the primary recommendation for advanced HCC, but their efficacy remains poor and other potential treatment modalities still need to be explored to further improve the survival of HCC patients (10). Most studies have shown that RFA for HCC less than 5 cm can achieve similar outcomes as surgery (11, 12). However, due to the limitations of RFA, it is not applicable to HCC larger than 5 cm. Radiotherapy and TACE are recommended as potential local treatment modalities for HCC larger than 5 cm. Nevertheless, the optimal local treatment modality for this tumor size remains controversial (13).

With the advancement of treatment techniques, external beam radiation therapy (EBRT) modalities have been developed and applied to locally advanced HCC with good results, including intensity-modulated radiotherapy (IMRT), stereotactic body radiotherapy (SBRT), and gamma knife radiosurgery (GKR) (14–16). EBRT has been shown to reduce the risk of liver failure by preserving the surrounding normal tissue while maintaining a high radiation dose (17, 18). A retrospective study by Li et al. found no difference in 1- and 5-year median progression-free survival (mPFS) (53.9, 7.5% vs. 54.5, 9.6%, *p* = 0.744) and mOS (73.5, 7.6% vs. 72.4, 13.2%, *p* = 0.151) between IMRT and SBRT for advanced HCC (15). In addition, in a meta-analysis, Chen et al. demonstrated that EBRT combined with sorafenib for inoperable HCC prolonged mPFS and mOS to 8.2 months and 19.2 months in HCC, respectively (15). Furthermore, a previous study also found a mOS of 20.1 months for advanced HCC patients treated with IMRT in combination with PD-1 inhibitors and anti-angiogenic therapy (3). TACE has also shown great promise as a treatment option for HCC. Numerous studies have confirmed that TACE is a safe and effective treatment option for HCC as long as the tumor’s donor artery can be isolated (19, 20). The latest study showed that advanced HCC patients undergoing TACE + lenvatinib had an mPFS of 10.6 months and an mOS of 17.8 months (21). A retrospective study of unresectable HCC also showed that TACE + pembrolizumab + lenvatinib could improve mOS to 18.1 months (22). Therefore, both EBRT and TACE have shown great promise in the treatment of HCC.

Although non-diffuse HCC with tumor diameter ≥ 5 cm is an indication for both EBRT and TACE (13), there is currently a lack of head-to-head studies comparing the efficacy of these two regimens to provide evidence for informed clinical decisions. Therefore, we conducted this multicenter research to compare the outcomes of EBRT and TACE for non-diffuse HCC with tumor diameter ≥ 5 cm.

## 2 Materials and methods

### 2.1 Patients

We retrospectively and consecutively searched the medical record systems of five Chinese tertiary hospitals to identify all HCC patients from May 11, 2012 to November 5, 2022. Subsequently, 1210 HCC patients who met the inclusion and exclusion criteria were included in our study.

The enrollment criteria for this study were as follows: 1) clinically or pathologically confirmed HCC; 2) Child-Pugh A/B; 3) tumor diameter ≥ 5 cm; 4) tumor number ≤ 5; 5) Eastern Cooperative Oncology Group score 0-2; 6) EBRT or TACE as the primary local treatment modality. The exclusion criteria were as follows: 1) diffuse HCC; 2) incomplete clinical data; 3) concurrent combination of other malignancies; 4) patients received local treatment other than EBRT and TACE; 5) hepatic encephalopathy or refractory ascites.

Patients were divided into EBRT group (n = 302) and TACE group (n = 908) according to the interpretation by clinicians for all patients who met the treatment criteria. The final choice of treatment option for patients was made after consideration of cost, patient preference, and medical evidence. In addition, we performed subgroup analysis for different tumor diameters (5-7 cm, 7-10 cm, and ≥ 10 cm).

This retrospective study was approved by the Ethics Committees of the Affiliated Hospital of Southwest Medical University and complied with the standards of the Declaration of Helsinki. The ethics committee abandoned the informed consent form because it was a retrospective study.

### 2.2 Treatment protocol

#### 2.2.1 EBRT

EBRT has been described in detail in previous studies (3, 16). Briefly, radiologists depicted tumor lesions under the guidance of computed tomography (CT) scans using a radiation planning system. The gross target volume primarily included the primary tumor lesion, whereas the clinical target volume (CTV) included the tumor lesion and subclinical lesions and the extent of possible infiltration. The planning target volume (PTV) was defined as a 5-10 mm outward from the CTV margin. The median radiation doses for GKR, IMRT, and SBRT were 42Gy (range 39-42 Gy), 45Gy (range 15-66 Gy), and 48Gy (range 30-61 Gy), respectively. A minimum of 95% of the PTVs was exposed to the prescribed doses, with 45 Gy, 30 Gy, 54 Gy, and 55 Gy, as the dose limits for the spinal cord, normal liver, stomach and duodenum, and colon, respectively.

#### 2.2.2 TACE

TACE has been described in detail in a previous study (16). Briefly, TACE was performed under a digital subtraction angiography machine. The Seldinger technique was employed to access the celiac trunk and superior mesenteric artery for catheter angiography to define the tumor size and blood supplying arteries. A microcatheter was then inserted into the tumor-supplying aorta and perfused with one or more chemotherapeutic agents (raltitrexed, cisplatin, mitomycin, and fluorouracil) and embolic agents (iodinated oil emulsion and gelatin sponge). The catheter and sheath were removed after embolization was confirmed to be successful by angiography, and pressure bandages were applied to puncture site to stop the bleeding.

### 2.3 PD-1 inhibitors and targeted drugs

Clinicians recommended PD-1 inhibitors (pembrolizumab, camrelizumab, and sintilimab, etc.) and targeted agents (sorafenib, lenvatinib, and bevacizumab, etc.) to the patients and made the final treatment decision with the patient’s consent. The dose of the drug was determined based on the height and weight of the patient.

### 2.4 Follow-up

After the procedure, patients were followed up with laboratory tests and Magnetic Resonance Imaging (MRI)/CT every 2-3 months. Laboratory tests included blood cell analysis, liver function [alanine aminotransferase (ALT), alkaline phosphatase (ALP), albumin, and aspartate aminotransferase (AST)], alpha fetoprotein (AFP), and pro-thrombin time. OS was defined as the time interval between the start of treatment and the date of patient death or final follow-up as the primary endpoint of the study.

### 2.5 Statistical analysis

χ2 and McNemar test were used to analyze categorical variables. One-to-one propensity score matching (PSM) was used to identify two groups (EBRT and TACE groups) with similar baseline characteristics. Matching variables included extrahepatic metastasis, sex, tumor number, Child-Pugh score, tumor size, age, leukocyte, ALT, AFP, platelet, ALP, portal vein tumor thrombus (PVTT), drinking history, HCV, HBV, lymph node metastasis, Barcelona Clinic Liver Cancer (BCLC) stage, targeted therapy, and PD-1 inhibitors. Survival curves for both groups were created using the Kaplan-Meier and compared using log-rank tests. Cox analysis was performed to detect indicators that influenced patient survival. All data in this study were analyzed using SPSS for Windows (version 26.0), and two tailed p < 0.05 was considered statistically significant.

## 3 Results

### 3.1 Patient characteristics

This study enrolled a total of 1210 HCC patients (EBRT group, 302; TACE group, 908) with tumor diameter ≥ 5 cm. In the EBRT group, 179, 101, and 22 HCC patients received GKR, IMRT, and SBRT, respectively. We followed the Cheng’s type of PVTT (16). Before matching, there were significant differences in tumor diameter, BCLC stage, PVTT, combined targeted drugs, and combined PD-1 inhibitors between the two patient groups (all *p* < 0.05). In total, 428 patients were identified using 1:1 PSM. After matching, we observed no difference in baseline characteristics between the two groups (**Table 1**).

**Table 1:** Baseline characteristics of the patients before and after PSM.

	Before PSM			After PSM		
Variable	EBRT	TACE	*P*	EBRT	TACE	*p*
Patients	302	908		214	214	
Male sex	260 (86.1)	764 (84.1)	0.415	183 (85.5)	185 (86.4)	0.888
Age ≥ 60 years	104 (34.4)	314 (34.6)	0.964	75 (35.0)	82 (38.3)	0.558
Child–Pugh score			0.718			0.192
5	130 (43.0)	399 (43.9)		101 (47.2)	91 (42.5)	
6	94 (31.1)	265 (29.2)		63 (29.4)	65 (30.4)	
7	48 (15.9)	151 (16.6)		36 (16.8)	39 (18.2)	
8	14 (4.6)	56 (6.2)		6 (2.8)	11 (5.1)	
9	16 (5.3)	37 (4.1)		8 (3.7)	8 (3.7)	
Number of tumors ≥ 2	217 (71.9)	691 (76.1)	0.140	162 (75.7)	168 (78.5)	0.451
Tumor diameter, cm			<0.001			0.974
≥ 5, < 7	108 (35.8)	212 (23.3)		63 (29.4)	65 (30.4)	
≥ 7, < 10	86 (28.5)	277 (30.5)		63 (29.4)	63 (29.4)	
≥ 10	108 (35.8)	419 (46.1)		88 (41.1)	86 (40.2)	
Serum AFP, ng/ml			0.386			0.078
< 200	137 (45.4)	389 (42.8)		93 (43.5)	100 (46.7)	
≥ 200, < 400	24 (7.9)	58 (6.4)		20 (9.3)	10 (4.7)	
≥ 400	141 (46.7)	461 (50.8)		101 (47.2)	104 (48.6)	
ALP levels ≥ 125 U/L	196 (64.9)	632 (69.6)	0.128	137 (64.0)	146 (68.2)	0.407
Platelet count ≥ 100 × 10^9^/L	232 (76.8)	700 (77.1)	0.923	170 (79.4)	161 (75.2)	0.342
ALT levels ≥ 40 U/L	170 (56.3)	509 (56.1)	0.943	120 (56.1)	120 (56.1)	1.000
Leukocyte ≥ 4 × 10^9^/L	256 (84.8)	753 (82.9)	0.457	188 (87.9)	172 (80.4)	0.052
Number of TACE ≥ 2	–	314 (34.6)		–	63 (29.4)	
Radiotherapy modalities						
GKR	179 (59.3)	–		146 (68.2)	–	
IMRT	101 (33.4)	–		58 (27.1)	–	
SBRT	22 (7.3)	–		10 (4.7)	–	
BCLC stage			<0.001			0.200
A	19 (6.3)	106 (11.7)		16 (7.5)	9 (4.2)	
B	30 (9.9)	181 (19.9)		27 (12.6)	28 (13.1)	
C	253 (83.8)	621 (68.4)		171 (79.9)	177 (82.7)	
Cheng’s type of PVTT	233 (77.2)	502 (55.3)	<0.001	156 (72.9)	164 (76.6)	0.494
I-II	109 (36.1)	282 (31.1)		86 (40.2)	89 (41.6)	
III-IV	124 (41.1)	220 (24.2)		70 (32.7)	75 (35.0)	
Etiology						
HBV	196 (64.9)	546 (60.1)	0.140	135 (63.1)	131 (61.2)	0.762
HCV	2 (0.7)	20 (2.2)	0.083	6 (2.8)	6 (2.8)	1.000
Alcohol	118 (39.1)	321 (35.4)	0.244	83 (38.8)	74 (34.6)	0.431
Lymph node metastasis	82 (27.2)	251 (27.6)	0.869	57 (26.6)	63 (29.4)	0.581
Extrahepatic metastases	76 (25.2)	225 (24.8)	0.893	55 (25.7)	65 (30.4)	0.308
Lung	37 (12.3)	145 (16.0)		28 (13.1)	44 (20.6)	
Bone	33 (10.9)	45 (5.0)		22 (10.3)	9 (4.2)	
Other	19 (6.3)	59 (6.5)		15 (7.0)	17 (7.9)	
Combined targeted drugs	138 (45.7)	210 (23.1)	<0.001	75 (35.0)	72 (33.6)	0.664
Combined PD-1 inhibitors	73 (24.2)	118 (13.0)	<0.001	32 (15.0)	21 (9.8)	0.061

PSM, propensity score matching; AFP, alpha fetoprotein; ALP, alkaline phosphatase; ALT, alanine aminotransferase; IMRT, intensity-modulated radiotherapy, SBRT, stereotactic body radiotherapy, GKR, gamma knife radiosurgery; BCLC, Barcelona Clinic Liver Cancer; PVTT, portal vein tumor thrombus; HBV, hepatitis B virus; HCV, hepatitis C virus; PD-1, programmed death 1; EBRT, External beam radiation therapy; TACE, transcatheter arterial chemoembolization.

### 3.2 OS

**Figure 1:**
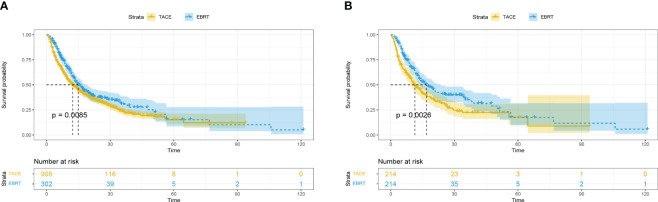
Overall survival in the EBRT and TACE groups before **(A)** and after **(B)** propensity score matching. EBRT, External beam radiation therapy; TACE, transcatheter arterial chemoembolization.

Before matching, 175 (57.9%) patients and 604 (66.5%) patients died in the EBRT and TACE groups, respectively. Compared with the TACE group, the EBRT group had a longer mOS [14.9 (95%CI 12.5-17.3) vs. 12.3 (95%CI 10.6-14.0) months]. Furthermore, the 1-, 3- and 5-year survival rates (59.4%, 30.3%, 14.5% vs. 50.2%, 23.2%, 14.0%; *p* = 0.0085) in the EBRT group were better compared with the TACE group (**Figure 1A**).

After matching, compared with the TACE group, the mOS in the EBRT group months was longer [16.8 (95%CI 12.5-21.1) vs. 11.4 (95%CI 8.7-14.1) months]. In addition, the 1-, 3- and 5-year survival rates (60.5%, 34.7%, 16.5% vs. 47.2%, 21.9%, 13.5%; *p* = 0.0026) observed in the EBRT group remained superior compared with the TACE group (**Figure 1B**).

### 3.3 Factors associated with OS

We performed Cox analysis after matching to determine the mortality risk factors for patients. Univariate analysis revealed that Child-B, tumor number ≥ 2, tumor diameter ≥ 10 cm, AFP ≥ 400 ng/ml, ALP ≥ 125 U/L, platelet ≥ 100000/μL, ALT ≥ 40 U/L, worse BCLC stage, PVTT, and TACE were significant risk factors for death in this patient population. The multivariable analysis showed that Child-A, ALP < 125 U/L, and EBRT were independent prognostic indicators for longer survival (**Table 2**).

**Table 2:** Univariate and multivariate Cox regression analysis of overall survival after PSM.

	Univariable Cox regression			Multivariable Cox regression		
Variable	HR	95%CI	p	HR	95%CI	p
Sex (male/female)	0.904	0.638-1.280	0.569			
Age (≥60/<60 years)	1.012	0.789-1.299	0.924			
Child-Pugh class (B/A)	2.066	1.574-2.712	<0.001	1.614	1.193-2.184	0.002
Number of tumor (≥2/<2)	1.504	1.112-2.035	0.008	1.254	0.883-1.780	0.206
Tumor diameter (≥10/<10 cm)	1.698	1.327-2.174	<0.001	1.185	0.905-1.550	0.217
AFP (≥400/<400 ng/ml)	1.457	1.141-1.859	0.003	1.111	0.854-1.446	0.432
ALP (≥125/<125 U/L)	2.054	1.567-2.694	<0.001	1.494	1.105-2.021	0.009
Platelet (≥100000/<100000/μL)	1.401	1.035-1.896	0.029	1.326	0.966-1.820	0.081
ALT (≥40/<40U/L)	1.565	1.219-2.010	<0.001	1.171	0.894-1.534	0.251
Leukocyte (≥4000/<4000/μL)	1.038	0.743-1.450	0.827			
BCLC			<0.001			0.468
A	1.000			1.000		
B	1.274	0.665-2.441	0.465	0.914	0.435-1.921	0.813
C	2.643	1.493-4.680	0.001	1.340	0.621-2.893	0.456
Cheng’s type of PVTT	2.138	1.585-2.885	<0.001	1.147	0.685-1.919	0.603
HBV (positive/negative)	1.192	0.927-1.533	0.170			
HCV (positive/negative)	1.300	0.690-2.447	0.417			
Alcoholism (positive/negative)	1.151	0.898-1.476	0.266			
Lymph node metastasis (yes/no)	1.288	0.987-1.681	0.062			
Extrahepatic metastases (yes/no)	1.247	0.957-1.625	0.103			
Treatment (EBRT/TACE)	0.689	0.540-0.880	0.003	0.705	0.551-0.902	0.005

PSM, propensity score matching; HR, hazard ratio; AFP, alpha fetoprotein; ALP, alkaline phosphatase; ALT, alanine transaminase; BCLC, Barcelona Clinic Liver Cancer; PVTT, portal vein tumor thrombus; HBV, hepatitis B virus; HCV, hepatitis C virus; EBRT, External beam radiation therapy; TACE, transcatheter arterial chemoembolization.

### 3.4 Subgroup analysis

**Figure 2:**
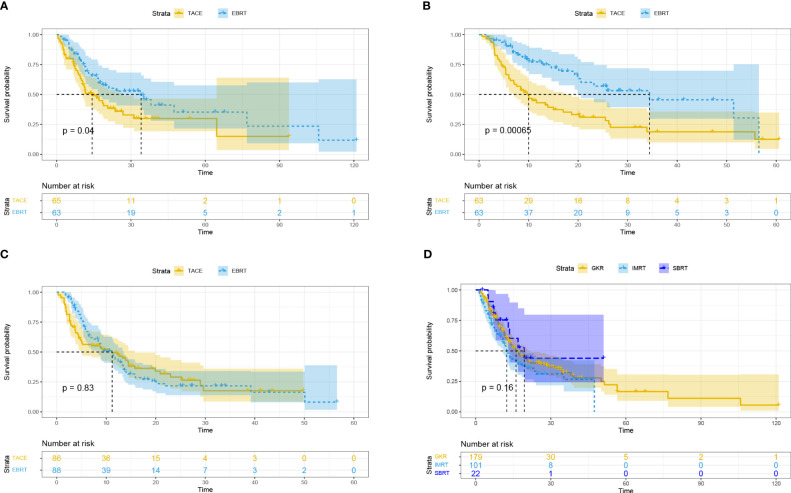
Overall survival based on different tumor diameters: ≥ 5, < 7 cm **(A)**; ≥ 7, < 10 cm **(B)**; ≥ 10 cm **(C)**. Overall survival for different radiotherapy modalities **(D)**. EBRT, External beam radiation therapy; TACE, transcatheter arterial chemoembolization; IMRT, intensity-modulated radiotherapy; SBRT, stereotactic body radiotherapy; GKR, gamma knife radiosurgery.

Subsequently, we performed subgroup analysis for different tumor diameters (5-7 cm, 7-10 cm, and ≥ 10 cm). The results showed that compared with the TACE group, the EBRT group had a better mOS for HCC with tumor diameter of 5-7 cm (34.1 vs. 14.3 months, *p* = 0.04; **Figure 2A**) and 7-10 cm (34.4 vs. 10 months, *p* = 0.00065; **Figure 2B**), but was not significantly different between the two groups for HCC with tumor diameters ≥ 10 cm (11.2 vs. 11.2 months, *p* = 0.83; **Figure 2C**).

Subgroup analysis based on different radiotherapy modalities (GKR, n = 179; IMRT, n = 101; SBRT, n = 22) revealed no differences in mOS among these three groups (16.1 vs. 12.4 vs. 19.4 months, *p* = 0.16; **Figure 2D**).

**Figure 3:**
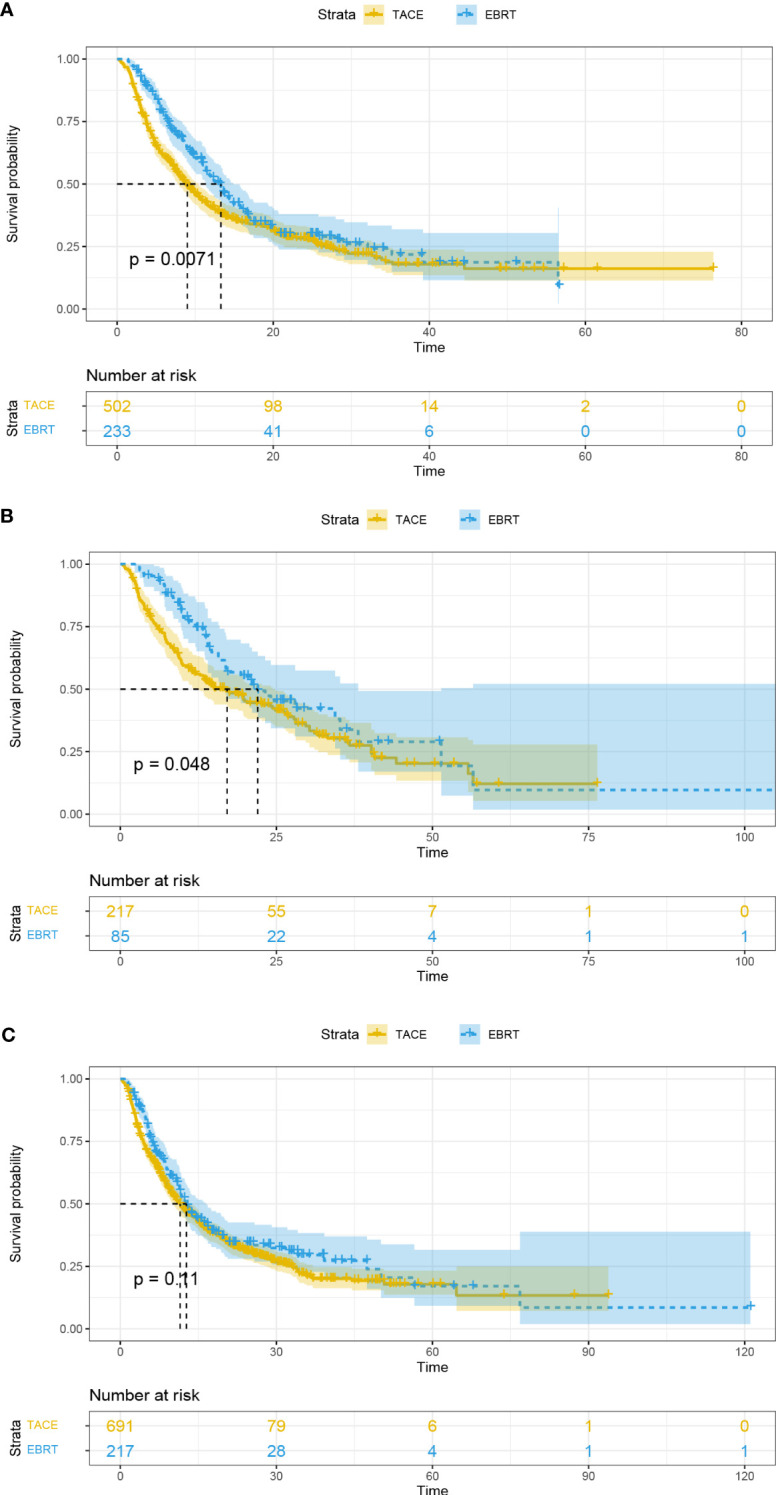
Overall survival curves for the patients with portal vein tumor thrombus **(A)**, tumor number < 2 **(B)**, and tumor number ≥ 2 **(C)**. EBRT, External beam radiation therapy; TACE, transcatheter arterial chemoembolization.

In addition, patients with PVTT and tumor number < 2 in the EBRT group exhibited longer OS than the TACE group (13.3 vs. 9 months, *p* = 0.0071, **Figure 3A**; 22.0 vs. 17.1 months, *p* = 0.048, **Figure 3B**; respectively). However, the mOS of the two groups was similar for patients with tumor number ≥ 2 (12.7 vs. 11.5 months, *p* = 0.11; **Figure 3C**).

## 4 Discussion

“A+T” regimen (atezolizumab+ bevacizumab) shows a great promise for HCC but its objective response rate (ORR) of 27.3% is still poor (10, 23). Therefore, it is of great clinical value to explore other local treatments with better efficacy in HCC. TACE and EBRT are both recommended treatment options in the National Comprehensive Cancer Network (NCCN) guidelines for non-diffuse HCC with tumor diameter ≥ 5 cm (13). However, despite these recommendations, there is currently a lack of a head-to-head study comparing the effectiveness of these treatments. Consequently, it is unclear which local treatment option is more appropriate for non-diffuse HCC with tumor diameter ≥ 5 cm.

In this study, both the pre- and post-matched EBRT groups had significantly longer mOS (14.9 vs. 12.3, *p* = 0.0085; 16.8 vs. 11.4, *p* = 0.0026, respectively) compared with the TACE group. In our further analysis, EBRT was more effective than TACE for HCC with tumor diameters of 5-7 cm and 7-10 cm. However, there was no difference in mOS for HCC with tumor diameter ≥ 10 cm between the two groups.

These results may be attributed to differences in the nature of TACE and EBRT treatments. Radiotherapy can reduce the risk of liver failure by preserving the surrounding normal tissue while maintaining a high radiation dose (17, 18). It not only maintains high local control but also stimulates immunogenic death, improves the tumor microenvironment, and promotes the expansion of anti-tumor T cells (24–26). However, advanced HCC is often associated with high tumor burden and complex blood supply. Tumors are prone to incomplete necrosis after embolization, which induces vascular endothelial growth factor expression and tumor revascularization and eventually leads to recurrence (20, 21). These factors may have contributed to the similarity in mOS of the two groups for HCC with tumor diameter ≥ 10 cm.

Currently, treatment options for HCC are still undergoing clinical trials. Finn and his colleagues (27) conducted a phase Ib study and found that lenvatinib + pembrolizumab improved the outcome of inoperable HCC (mPFS = 9.3 months, mOS = 22 months). Furthermore, in the ORIENT-32 study of inoperable HCC, mPFS (4.6 vs. 2.8 months, *p* < 0.001) and mOS (not reached vs. 10.4 months, *p* < 0.001) were observed to be longer with sintilimab-bevacizumab compared with sorafenib (7). In addition, Li et al. (15) found no difference in mOS (10 vs. 8 months, *p* = 0.165) between HCC patients with PVTT receiving SBRT and those receiving IMRT. Furthermore, a 12.1-month mOS was observed with selective internal radiotherapy + sorafenib in the treatment of advanced HCC (28). The LAUNCH research illustrated that lenvatinib + TACE had longer mPFS (10.6 vs. 6.4 months, *p* < 0.001) and mOS (17.8 vs. 11.5 months, *p* < 0.001) compared with lenvatinib for advanced HCC (21). In our study, EBRT exhibited superior efficacy compared with TACE in the treatment of non-diffuse HCC with tumor diameter ≥ 5 cm.

In our subgroup analysis, similar efficacy was found in the GKR group, the IMRT group, and the SBRT group. This result might be due to the smaller sample sizes in the three groups, which influenced the interpretation of the outcomes. Sure, SBRT and IMRT were also demonstrated to have similar efficacy in the treatment of HCC by Li et al. (15). However, there is a lack of head-to-head studies comparing GKR with other radiotherapy modalities. This is the first study to confirm that GKR has comparable outcomes for HCC to IMRT and SBRT.

In the Cox analysis, EBRT was identified as an independent predictor of OS. Moreover, Child-B and ALP ≥ 125 U/L predicted worse OS in patients. These indicators have also been previously reported to be correlated with poorer prognosis (29, 30).

The limitations of this study are equally significant. First, all potential confounders could not be completely eradicated, even after performing PSM. Second, the SBRT group had fewer patients, which could have affected the representation of our results. In addition, patients received different targeted and PD-1 inhibitors, which might have affected our interpretation of the results. Prospective studies are needed in the future to provide further evidence of the efficacy of EBRT.

## Conclusion

In conclusion, EBRT is more effective than TACE as the primary local treatment for HCC with tumor diameter ≥ 5 cm, especially for HCC with a tumor diameter of 5-10 cm. This finding lays the foundation for future studies on radiotherapy for HCC.

## Data availability statement

The original contributions presented in the study are included in the article/supplementary material. Further inquiries can be directed to the corresponding authors.

## Ethics statement

The studies involving humans were approved by the Affiliated Hospital of Southwest Medical University. The studies were conducted in accordance with the local legislation and institutional requirements. The ethics committee/institutional review board waived the requirement of written informed consent for participation from the participants or the participants’ legal guardians/next of kin because this retrospective study was approved by the Ethics Committee of the Southwest Medical University Hospital (approval number KY2020254) and complied with the standards of the Declaration of Helsinki. The Ethics Committee abandoned the informed consent form because it was a retrospective study.

## Author contributions

YH: Formal Analysis, Funding acquisition, Project administration, Writing – review & editing. KS: Data curation, Supervision, Validation, Writing – original draft. FW: Data curation, Supervision, Validation, Writing – original draft. XL: Data curation, Supervision, Validation, Writing – original draft. HC: Data curation, Supervision, Validation, Writing – review & editing. KH: Methodology, Software, Writing – review & editing. ZWa: Data curation, Writing – review & editing. LW: Data curation, Writing – review & editing. YS: Data curation, Writing – review & editing. JC: Data curation, Writing – review & editing. ZWu: Data curation, Writing – review & editing. YJ: Data curation, Writing – review & editing. HL: Data curation, Writing – review & editing. TG: Data curation, Writing – review & editing. CW: Data curation, Writing – review & editing. YL: Data curation, Writing – review & editing. ML: Data curation, Writing – review & editing. QG: Data curation, Writing – review & editing. KX: Project administration, Writing – review & editing. LG: Project administration, Writing – review & editing. JZ: Data curation, Formal Analysis, Writing – original draft.
